# Supplementary material for: Comprehensive Analysis of NAC Genes Reveals Differential Expression Patterns in Response to Pst DC3000 and Their Overlapping Expression Pattern during PTI and ETI in Tomato
Source: Genes (Basel). 2022 Nov 2;13(11):2015. doi: 10.3390/genes13112015 (PMC9690738; doi:10.3390/genes13112015)
Supplement: Supplementary file 1 [file genes-13-02015-s001.zip › Table S1.pdf]

Table S1. RT-qPCR primers of *SSLNAC* genes used in this study.

LoC	gene name	F	R
Solyc02g069960	*SSlNAC1*	TCCAAATAGTTCCCAGAT	AGTTGCCATGTTCTAACC
Solyc02g087920	*SSlNAC2*	AAGACCTCCGAGTCCATA	TCCTCAGCCAAACTAAAA
Solyc04g005610	*SSlNAC3*	TGGCTTCTAATGGACAAC	ATGGCTTGATGAGGCATA
Solyc04g009440	*SSlNAC4*	TGTGACAAGGAGGTTCAG	TGTCTTGGAAATTGTTGA
Soly05g007550	*SSlNAC5*	GTGGTGGTGGTGGTGTTC	CATCAGCAGCCATATTCA
Soly05g021090	*SSlNAC6*	TTGGGGATGCAAGTTCTA	TTTGTTGGTTCCTGGTTT
Solyc05g007770	*SSlNAC7*	TGATGACCAAGGTTACAC	TGAGATTGAAATGTTGGA
Solyc06g060230	*SSlNAC8*	ATTCAGTTCCGAAGATGC	GCTATTATCCACCGTTGTG
Solyc07g006840	*SSlNAC9*	GGAGGTGATTATGGTGAG	CAAAGTTGTTCGAGGTAG
Soly07g045030	*SSlNAC10*	GTTACTACAATAATAGCCA	GGCTAAATCAACGACAGA
Solyc07g063420	*SSlNAC11*	TGAAGGTGGATCACAATA	TGAGAAGACTTACGATAGAT
Soly07g63410	*SSlNAC12*	AGTTAAGTTTGAAGGAGG	CATATTTACTGTTTGGGTC
Solyc08g007020	*SSlNAC13*	CTAAGGCTGGATACTGGA	GAACTCATAGCGAGGACA
Solyc08g077110	*SSlNAC14*	CTTTCCCGAGTTTTAGAG	GATTTGGATTCCTGTTTG
Solyc06g061080	*SSlNAC15*	ATCCTTCAAAGGAATGTC	CGACTGAGAAGGGAGATA
Solyc10g06880	*SSlNAC16*	TGGAATAACGACGAAGCA	GAAATTGGCAATGGAGCT
Solyc10g083450	*SSlNAC17*	AAGACGAGTTGGAAGATG	TCGATAATTGGGAAAGTG
Solyc11g017470	*SSlNAC18*	TCAACAAACCCATCTTCG	TCGGCTTCGGCTCACTCT
Solyc11g065540	*SSlNAC19*	GGGCGGAGGTATGGAATC	CAAGAGGAAATGGTGGTG
Solyc12g013620	*SSlNAC20*	GTTCAGGGTCAGGCTCAG	CCCGCTTTCAACTTCTTC
β-actin	β-actin	GTCCTCTTCCAGCCATCCAT	ACCACTGAGCACAATGTTACCG
